# Inhibition of the ecto-beta subunit of F1F0-ATPase inhibits proliferation and induces apoptosis in acute myeloid leukemia cell lines

**DOI:** 10.1186/1756-9966-31-92

**Published:** 2012-11-09

**Authors:** Zhao Wen-Li, Wang Jian, Tao Yan-Fang, Feng Xing, Li Yan-Hong, Zhu Xue-Ming, Zhang Min, Ni Jian, Pan Jian

**Affiliations:** 1 Department of Hematology and Oncology, Children’s Hospital of Soochow University, Suzhou, China; 2 Translational Research Center, Second Hospital, The Second Clinical School, Nanjing Medical University, Nanjing, China

**Keywords:** Acute myeloid leukemia, Apoptosis, Ecto-ATPase β subunit, Proliferation, HL-60, MV4-11

## Abstract

**Background:**

Leukemia, a heterogeneous clonal disorder of hematopoietic progenitor cells, presents a world-wide health problem, especially in childhood. F1F0 ATPase, an inner mitochondrial enzyme, is expressed on the plasma membrane of tumor cells, and its inhibition induces both anti-angiogenic and anti-tumorigenic activity.

**Methods:**

Monoclonal Antibody (McAb) against ATPase was produced by polyethylene glycol-mediated fusions and screened by ELISA. Proliferation, cell cycle and apoptosis of cells were analyzed when the surface ATPase of cells was blockaded with McAb.

**Results:**

We detected cell-membrane expression of the F1F0 ATPase β subunit on 0.1% to 56% of the 11 cell lines derived from leukemia, including acute myeloid leukemia (AML). We produced a monoclonal antibody, McAb7E10, which recognizes both the native and recombinant ATPase β subunit, with a dissociation constant (KD) of 3.26E^–10^. We demonstrate that McAb7E10 binds to ATPase at the cell surface, where it is able to inhibit ATP synthesis. McAb7E10 significantly inhibited proliferation of AML cell lines *in vitro*: the relative inhibitory rates of 50 μg/mL McAb7E10 treated MV4-11and HL-60 cells were 69.6% and 81.9% respectively. Cell cycle analysis indicated that McAb7E10 significantly induced apoptosis in MV4-11 and HL-60 cells: the relative rates of apoptosis in 5, 10 and 50ug/mL McAb7E10 treated MV4-11 cells was 3.6 ± 0.83%, 8.4 ± 1.69% and 17.3 ± 2.56% compared to 1.5% ± 0.85% in mouse IgG treated cells (p < 0.01). The relative rate of apoptosis in 5, 10 and 50ug/mL McAb7E10 treated HL-60 cells was 5.5 ± 2.37%, 11.3 ± 3.62% and 19.9 ± 3.31% compared to 1.56% ± 0.97% in mouse IgG treated cells (p < 0.01). Annexin V staining demonstrated that the relative apoptotic rates in 50 μg/mL McAb7E10 treated MV4-11 and HL-60 cells were 50.5% ± 7.04% and 32.9% ± 4.52%, respectively, significantly higher than IgG control antibody treated cells were 21.9% ± 3.11% and 15.3% ± 3.95%, p < 0.01.

**Conclusions:**

These findings indicate that ectopic expression of ATPase β subunit may be a tumor-associated antigen in hematological malignancies. The F1F0 ATPase β subunit provides a potential target for immunotherapy in AML and hematological malignancies.

## Background

F1F0 ATP synthase is a complex molecular motor responsible for the majority of ATP synthesis in all organisms [1,2]. The ATP synthase β subunit is mostly expressed in the inner mitochondrial membrane of normal cells [3-9]. Over the last few years, reports by several independent groups have described the presence of various subunits of ATP synthase at the cell surface of mammalian cells, which have been termed ecto-F1F0-ATPase [5,10-13]. Recent studies have shown that the β-subunits of F1F0 ATPase are located on the plasma membrane, as well as within the mitochondrial membrane of human vascular endothelial cells and tumor cells [5,6,10,14]. Most of the cell lines which are reported to express ecto-F1F0-ATPase β-subunits are leukemia cell lines, including K562, Raji [15], Daudi, U937 [11], Jurkat [16], ST-Emo and Rma-S [17].

In endothelial cells, the ecto-F1F0-ATPase β subunit has been identified as a receptor for angiostatin, a naturally occurring inhibitor of angiogenesis [5,14] which inhibits endothelial cell proliferation, tube formation and migration. Several conflicting reports have debated whether ecto-F1F0-ATPase is functional in tumor cells [3,10,15,17-20]. Recent data has shown that the mitochondrial F1-ATPase is expressed on tumor cell surface and promotes tumor recognition by Vgamma9Vdelta2 T cells. [11]. T lymphocytes are known to participate in the immune response against various intracellular pathogens, including tumor cells. Additionally, other research has demonstrated that inhibition of the ecto-F1F0-ATPase β-subunit is directly cytotoxic to tumor cells [3,18,21]. This data indicates that identification of novel ecto-F1F0-ATPase β subunit inhibitors, with both anti-angiogenic and anti-tumorigenic activities, may confer a greater therapeutic advantage by affecting cancer cells via by multiple mechanisms with potentially additive effects.

In this study, we analyzed expression of the ecto-F1F0-ATPase β subunit in eleven cell lines derived from hematological malignancies and HUVECs, a positive control human vascular endothelial cell line. Most of cell lines derived from hematological malignancies expressed the ecto-F1F0-ATPase β subunit. We produced a monoclonal antibody 7E10 (McAb7E10) specific to the human F1F0 ATPase β subunit, which inhibited proliferation and induced significant apoptosis in the acute myeloid leukemia (AML) cell lines, MV4-11 and HL-60. These results suggest that the abnormal cell surface expression of the ecto-F1F0-ATPase β subunit may provide a potential target for cancer immunotherapy in hematological malignancies, particularly AML.

## Methods

### Cell culture

Cell lines derived from hematological malignancies (HL-60, MV4-11, U937, K562, Raji, and Jurkat) were obtained from the American Type Culture Collection (ATCC). SHI-1, MOLT4, DAMI, CCRF and 697 cell lines (gifts from Professor Wang Jian-Rong, The Cyrus Tang Hematology center of Soochow University). The entire cell lines were maintained at 37°C in the RPMI 1640 supplemented with 10% fetal bovine serum, 100 U/ml penicillin and 100 mg/ml streptomycin [22]. HUVEC cells were a gift from Professor Yang Zhi-Hua (Department of Cell and Molecular Biology, Cancer Institute, Chinese Academy of Medical Sciences, Beijing, China) and were cultured in M200 basal culture media supplemented with low serum growth supplement (Cascade Biologics, PL, USA), 100 U/ml penicillin and 100 mg/ml streptomycin [23-25]. All cells were cultured at 37°C in a 5% CO_2_ humidified atmosphere.

### Flow cytometric assay

Cells were collected, washed twice with phosphate buffered saline (PBS), adjusted to 1 × 10^6^ cells/ml, and incubated with ATP synthase subunit beta monoclonal antibody (1:300; MitoScience MS503, EA, USA) for 30 min at 4°C. After washing three times with PBS, fluorescein-isothiocyanate (FITC)-labeled goat anti-mouse IgG (Jackson,WG, PA) diluted in PBS was added, incubated for 20 min at 4°C, then cells were washed three times with PBS, 1 ug/ml PI(Propidium Iodide, Sigma, St. Louis, MO, USA)) was added to exclude the dead cells and membrane antigen expression was analyzed using a fluorescence-activated cell sorter (ESP Elite, Beckman Coulter, Fullerton, CA, USA). All experiments were performed three times.

### Production of functional F1F0 ATPase Î² subunit antibody

Six to eight weeks old female BALB/c mice were subcutaneously immunized with hATP5B (F1F0 ATPase β subunit) which had been expressed using a prokaryotic system, as previously described [3], and mixed with Freund’s complete adjuvant (Sigma, St. Louis, MO, USA). The antibody valences in peripheral blood were determined using an ELISA as Gou, L. T. described [21], and three days after the last boost, 5 × 10^8^ sensitized spleen cells were harvested, mixed and fused with 1 × 10^8^ SP2/0 myeloma cells, in 50% polyethylene glycol 1500 in a proportion of 8:1. The fused cells were plated in 96-well plates (6 × 10^5^/well) and cultured for two weeks in RPMI 1640 with 10% fetal calf serum containing hypoxanthine, aminopterin, and thymidine to select for positive hybrid cells. The positive hybridoma cells were subcloned by limiting dilution, and 10–12 week old female BALB/c mice were inoculated with 3 × 10^6^ hybridoma cells [3,26]. The antibodies were further purified from the ascites via Protein-A affinity chromatography [3]. The antibody with the highest valence against the F1F0 ATPase β subunit was named as McAb7E10 and used in further experiments.

### Western blotting and BIAcore analysis

Cellular proteins were extracted in 40 mM Tris–HCl (pH 7.4) containing 150 mM NaCl and 1% (v/v) Triton X-100 and supplemented with a cocktail of protease inhibitors. Equal amounts of protein were resolved on 12% SDS-PAGE gels then transferred to a PVDF membrane. After blocking with 5% non-fat milk, the membranes were incubated with McAb7E10 antibody overnight at 4°C, then with HRP-conjugated sheep anti-mouse IgG secondary antibody (Vector, Burlingame, CA, USA). After washing, the protein bands were visualized using the SuperEnhanced chemiluminescence detection kit (Applygen Technologies Inc., Beijing, China) and X-ray film (Kodak,NY,USA). The binding and dissociation kinetics of McAb7E10 with the recombinant ATPase β subunit were determined using a BIAcore surface plasmon resonance instrument (Pharmacia, Uppsala, Sweden) [27-31]. Briefly, 1400 RU of the recombinant ATPase β subunit (25 ug/mL in 10 mmol/L sodium acetate, pH 4.5) were covalently bound through amino groups to a CM5 sensor chip [32-34].

### ATPase activity assay

1*10^4^ cells per well were equilibrated with serum-free medium at 37°C with 5% CO_2_ overnight, respectively, in 96-well plates. Then the cells were treated with different concentrations of McAb7E10, oligomycin (Sigma, St. Louis, MO, USA), a known inhibitor of ATPase F1 or mouse IgG for 30 min. The cells were then incubated with adenosine diphosphate (Sigma, St. Louis, MO, USA) for 60 s, and supernatants were removed and assayed for ATP production using a bioluminescence assay kit (Invitrogen, Carlsbad, CA, USA). Samples were injected with the ATP assay mixture (Promega, Madison, WI, USA) and incubated for 10 min to stabilize the luminescence signal. Recordings were made in an Analyst HT (Molecular Devices, Sunnyvale, CA, USA) over a 20 s period. Data are expressed as moles of ATP per well based on standards determined under the same conditions during each experiment.

### Cell proliferation assay

Acute myeloid leukemia (AML) cells (MV4-11 and HL-60) were seeded in 96-well plates at 50,000 cells per well and 5–50 ug/mL mouse control IgG or 5–50 ug/mL McAb7E10 antibody was added. After 24, 48, 72, 96 or 120 h, 20 μL 5 mg/ml MTT (3-(4,5-dimethylthiazol-2-yl)-2,5- diphenyltetrazolium bromide) solution was added to each well, incubated at 37°C for 4 h, then the media was removed and 200 μL dimethylsulfoxide (DMSO) was added. Optical density (OD) values were measured at 490 nm using a scanning multi-well spectrophotometer (BioRad Model 550, Hercules, CA, USA), and the survival rates of McAb7E10 treated cells were calculated relative to the control antibody treated cells. All experiments were performed in triplicate and repeated twice. The results were analyzed using ANOVA and the Student-Newman-Keuls tests, *p* < 0.05 were considered significant.

### Cell cycle analysis

Cells were harvested and a single cell suspension was prepared in buffer (PBS + 2% FBS), washed twice and adjusted to 1 × 10^6^ cells/ml. Aliquots of 1 ml cell suspension were placed in 15 ml polypropylene V-bottomed tubes and 3 ml cold absolute ethanol was added to fix the cells for at least 1 h at 4°C. Cells were washed twice in PBS, 1 ml propidium iodide staining solution was added to the cell pellet, mixed well, and 50 μl RNAse A stock solution was added and incubated for 3 h at 4°C before flow cytometry analysis was performed.

### Cell apoptosis analysis

Cell apoptosis was analyzed using the Annexin V-FITC Apoptosis Detection Kit (Cat. No 556570; BD Franklin Lakes, NJ, USA) according to the manufacturer’s instructions. Briefly, incubated with mouse IgG or McAb7E10 antibody for 48 hours, then cells were washed twice with cold PBS, resuspended in 1x Binding Buffer at 1 × 10^6^ cells/ml and a 100 μl (1 × 10^5^ cells) aliquot was transferred to a 5 ml culture tube. 5 μl Annexin V and 10 μl vital dye was added, gently mixed, incubated for 15 min at RT in the dark, then 400 μl of 1x Binding Buffer was added to each tube and immediately analyzed by flow cytometry. All experiments were performed three times.

### Statistical analysis

All data are presented as mean ± SD. Statistical analysis was performed using SPSS statistical software (SPSS Inc, Chicago, IL, USA), *p* ≤ 0.05 were considered significant.

## Results and discussion

### The ecto-ATPase β subunit is expressed in cell lines from hematologic malignancies

**Figure 1:**
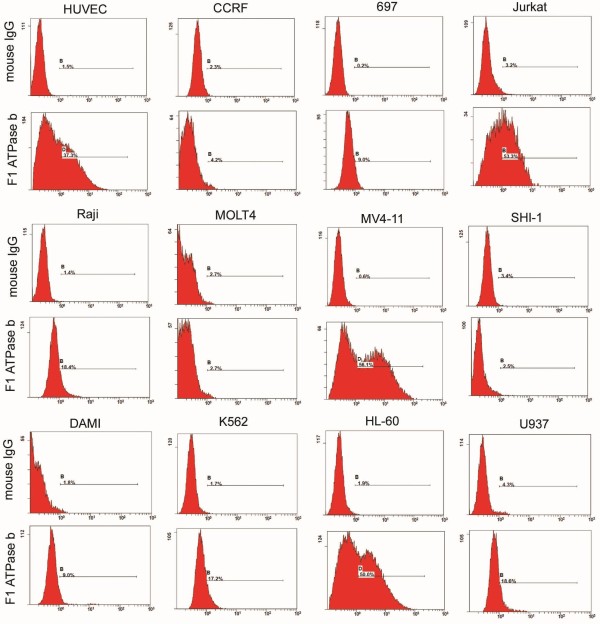
**Expression of ecto-ATPase β subunit in cell lines from hematological malignancies.** Cells were collected, incubated with an ATP synthase subunit β monoclonal antibody or mouse IgG control antibody, then with fluorescein-isothiocyanate (FITC)-labeled goat anti-mouse IgG and membrane ATP synthase subunit β expression was analyzed using fluorescence activated cell sorting (FACS). FACS results of 11 leukemia cells and HUVEC cells incubated with control IgG and ATP synthase subunit β monoclonal antibody.

The ATP synthase β subunit is known to be constitutively expressed in the inner mitochondrial membrane of normal cells, and ectopically expressed in primary cultured endothelial cells [3-7]. Liver carcinoma cells and lung carcinoma cells also express the ATP synthase β subunit on their cell surface [18,21]. In this study, we found that the ATP synthase β subunit is upregulated and ectopically expressed on the cell surface of human AML cells. Using flow cytometry, the β subunit of F1F0 ATPase was detected in 11 leukemia cell lines (two ALL cell lines 697 and Jurkat; three lymphoma cell lines CCRF, Raji and MOLT4; six myeloid leukemia cell lines MV4-11, SHI-1,DAMI, K562,HL-60 and U937). MV4-11, HL-60 and Jurkat are the top three cells (Figure 1). The β subunit of F1F0 ATPase was also detected in the positive control HUVEC cell line (Figure 1). The number of cells expressing ecto-ATPase β subunit on the cell membrane ranged from 0.1% to 56%. The percentage of cells expressing ecto-ATPase β subunit on the cell membrane in the K562 cell line (17.2%), derived from a 53 year old female CML patient, and the monocytic cell line U937 (18.6%), were similar to the previous report of *Scotet E* et al. [11].

### Production and characterization of McAb7E10

**Figure 2:**
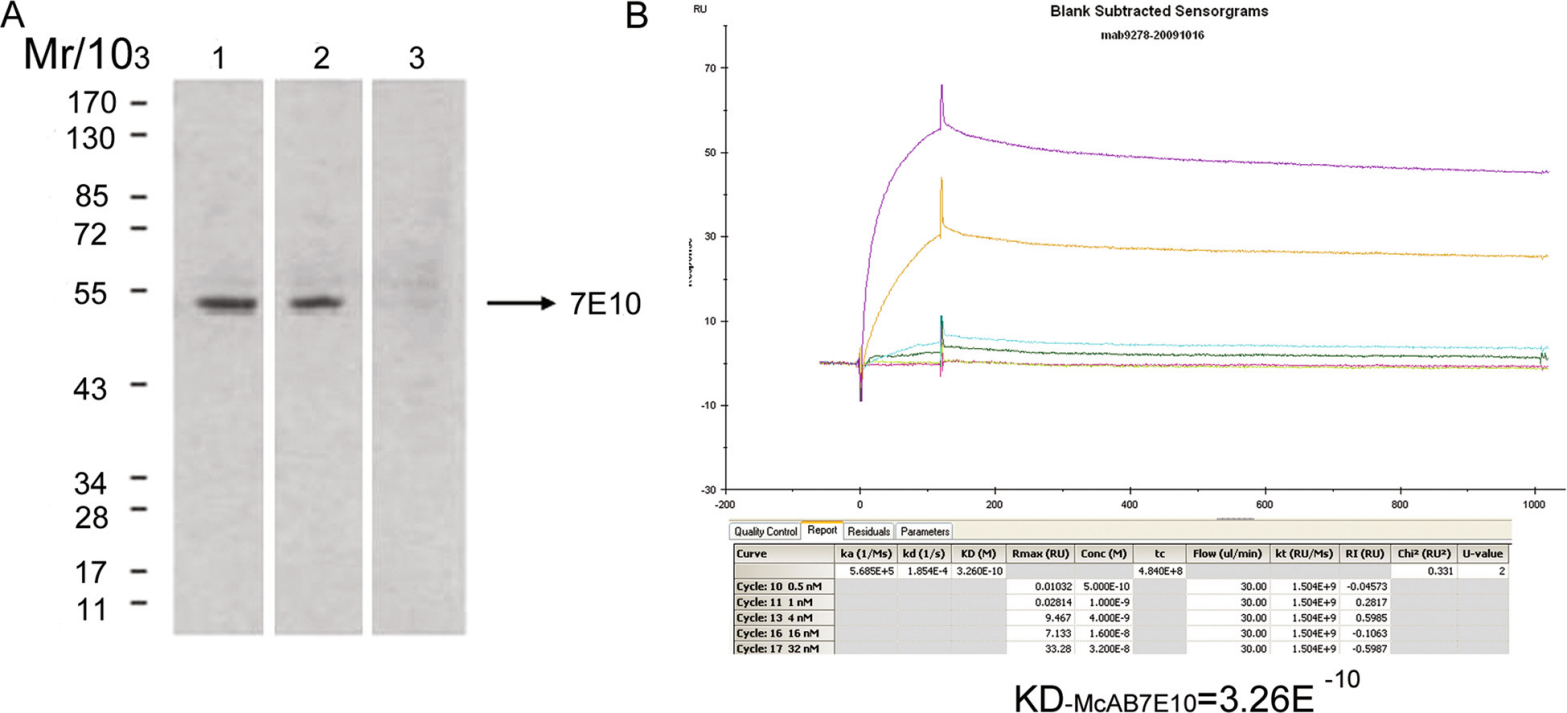
**Production and characterization of McAb7E10.** A monoclonal antibody with a high valency against F1F0 ATPase β subunit was developed and named McAb7E10. (**A**) In Western blot analysis, the McAb7E10 antibody detected a single immunoreactive band in HUVEC protein lysate (lane 1) and recombinant ATPase β subunit protein (lane 2), but did not detect recombinant human ATPase α subunit protein (lane3). (**B**) The affinity of McAb7E10 to recombinant ATPase β subunit was evaluated using BIAcore. The affinity of McAb7E10 to the recombinant ATPase β subunit was evaluated using BIAcore, and the dissociation constant was KD_McAb7E10_ = 3.26E^–10.^

In order to generate a monoclonal antibody (McAb) against the natural epitopes of the ATPase catalytic subunit, we immunized BALB/c mice with both natural immunogen and the human ATPase β subunit, which had been expressed in prokaryotes. After several fusion experiments, hundreds of monoclonal hybridoma cells were obtained. One immunoglobulin G1 (IgG1) hybridoma clone, named McAb7E10, recognized both the native and recombinant ATPase β subunit. In Western blot analysis, the McAb7E10 antibody identified a single band corresponding to the molecular mass of the ATPase β subunit, and did not cross react with the ATPase α subunit (Figure 2A). The affinity of McAb7E10 to the recombinant ATPase β subunit was evaluated using BIAcore, and the dissociation constant was KD_McAb7E10_ = 3.26E^–10^ (Figure 2B), which is higher than the KD of 4.24E^–9^ of the previously characterized ATPase β subunit antibody McAb178-5 G10 [3].

### McAb7E10 inhibits cell surface ATP generation in AML cells

**Figure 3:**
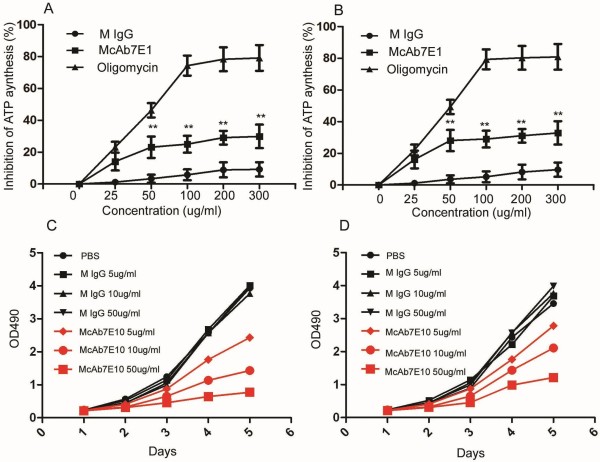
**McAb7E10 inhibits cell surface ATP generation and proliferation in AML cell.** To examine the inhibitory effect of the antibody on ATP synthesis, a cell surface ATP generation assay was performed. Results showed that McAb7E10 antibody significantly inhibited ATP synthesis in AML cells. The effect of McAb7E10 on the proliferation of the AML cell lines MV4-11 and HL-60 was evaluated using the MTT assay. (**A**, **B**) ATP generation on the surface of MV4-11 (A) and HL-60 (B) cells is inhibited dose-dependently in the presence of McAb7E10 and oligomycin. Oligomycin, a known inhibitor of ATP synthase F1, was used as positive control and mouse IgG as negative control. Data represent means ± SD. (**C**) Proliferation analysis of MV4-11 cells treated with mouse IgG and McAb7E10. At 120 h, the relative inhibitory rates for 5, 10 and 50 μg/mL McAb7E10 treated MV4-11 cells were 24.5%, 44% and 69.6% respectively, compared to control mouse IgG treated cells. (**D**) Proliferation analysis of HL-60 cells treated with mouse IgG and McAb7E10. At 120 h, the relative inhibitory rates for 5, 10 and 50 μg/mL McAb7E10 treated HL-60 cells were 39.4%, 62.1% and 81.9% respectively, compared to control mouse IgG treated cells.

To examine the inhibitory effect of the antibody on ATP synthesis, a cell surface ATP generation assay was performed. Results showed that McAb7E10 antibody significantly inhibited ATP synthesis in AML cells. The relative inhibitory rates in 25, 50 and 100 ug/mL McAb7E10 treated MV4-11 cells were 14.1%, 23.1% and 25.0%, in HL-60 cells were 16.1%, 28.1% and 29.3% respectively (Figure 3A, 3B). The maximal inhibition of McAb7E10 to MV4-11 and HL-60 cells was ∼30% (300 μg/mL), and the maximal inhibition of oligomycin to both cells was ∼80% (300 μg/mL).

### McAb7E10 inhibits AML cell proliferation and induces apoptosis in AML cells

This study provides evidence that McAb7E10 preferentially binds to the cell surface ATPase β subunit, and can inhibit cell proliferation and induce apoptosis in MV4-11 and HL-60 AML cells. The effect of McAb7E10 on the proliferation of MV4-11 and HL-60 cells was evaluated using the MTT assay. Compared to control mouse IgG treated cells, after 120 h, the relative inhibitory rates in 5, 10 and 50 ug/mL McAb7E10 treated MV4-11 cells were 24.5%, 44% and 69.6%, respectively (Figure 3C). After 120 h, the relative inhibitory rates in 5, 10 and 50 ug/mL McAb7E10 treated HL-60 cells were 39.4%, 62.1% and 81.9%, respectively (Figure 3D). These results indicate that McAb7E10 can significantly inhibit the proliferation of AML cells *in vitro*.

**Figure 4:**
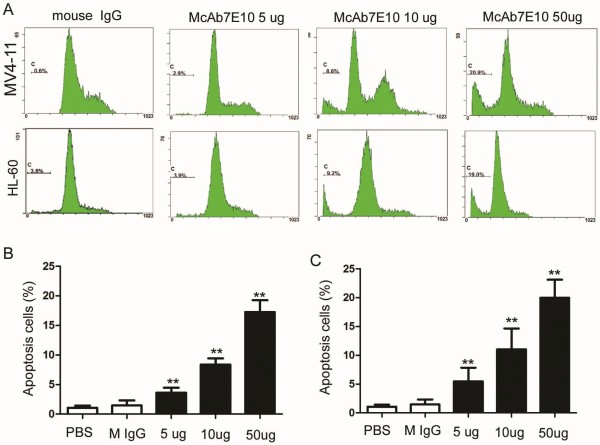
**Analysis of effect of McAb7E10 on the cell cycle in AML cell lines.** Cells were harvested, fixed, stained with propidium iodide staining and analyzed by flow cytometry. (**A**) Cell cycle analysis results of MV4-11 and HL-60 cell treated with different dose of McAb7E10. (**B**) The relative rate of apoptosis in 5, 10 and 50 ug/mL McAb7E10 treated MV4-11 cells was 3.6 ± 0.83%, 8.4 ± 1.69% and 17.3 ± 2.56% compared to 1.5% ± 0.85% in mouse IgG treated cells, *p* < 0.01. (**C**) The relative rate of apoptosis in 5, 10 and 50 ug/mL McAb7E10 treated HL-60 cells was 5.5 ± 2.37%, 11.3 ± 3.62% and 19.9 ± 3.31% compared to 1.56% ± 0.97% in mouse IgG treated cells, *p* < 0.01.

**Figure 5:**
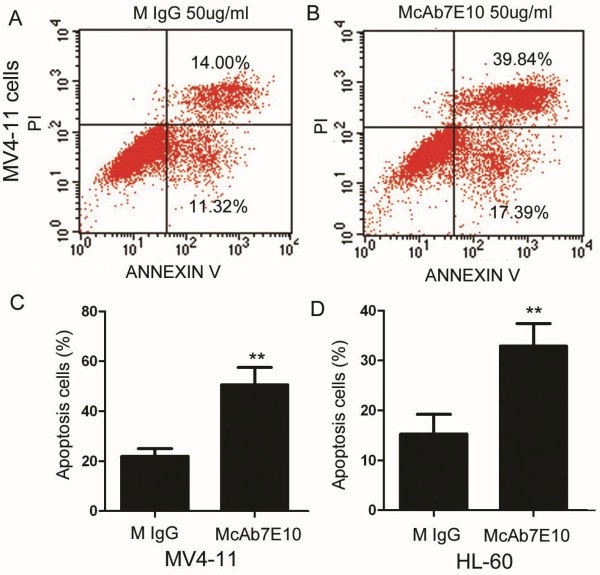
**McAb7E10 induces apoptosis in AML cell lines.** (**A**, **B**) Annexin V staining and flow cytometry was used to confirm that McAb7E10 induced apoptosis in AML cells. (**C**) The relative rate of apoptosis in 50 μg/ml McAb7E10 treated MV4-11 cells was 50.5% ± 7.04% vs 21.9% ± 3.11% in mouse IgG treated cells, *p* < 0.01. (**D**) The relative rate of apoptosis in 50 μg/ml McAb7E10 treated HL-60 cells was 32.9% ± 4.52% vs 15.3% ± 3.95% in mouse IgG treated cells, *p* < 0.01.

Using cell cycle analysis and Annexin V staining, a subpopulation of cells before the G1 population was detected after treatment with McAb7E10, indicating cells with abnormal nuclei which can be considered to be apoptotic and dead cells. The relative rate of apoptosis in 5, 10 and 50 ug/mL McAb7E10 treated MV4-11 cells was 3.6 ± 0.83%, 8.4 ± 1.69% and 17.3 ± 2.56% compared to 1.5% ± 0.85% in mouse IgG treated cells (*p* < 0.01, Figure 4A, 4B). The relative rate of apoptosis in 5, 10 and 50 ug/mL McAb7E10 treated HL-60 cells was 5.5 ± 2.37%, 11.3 ± 3.62% and 19.9 ± 3.31% compared to 1.56% ± 0.97% in mouse IgG treated cells (*p* < 0.01, Figure 4A, 4C). To determine whether McAb7E10 can induce apoptosis of leukemia cells, we test the apoptosis of cells with Annexin V test Kit. The data showed that the relative apotosis rate of 50ug/ml McAb7E10 treated MV4-11 cells was 50.5% ± 7.04% vs mouse IgG treated cells was 21.9% ± 3.11% P < 0.01 (Figure 5 A-C). The relative apotosis rate of 50ug/ml McAb7E10 on HL-60 cells was 32.9% ± 4.52% vs mouse IgG treated cells was15.3% ± 3.95% *P < 0.01* (Figure 5D).

### ATPase Î² subunit inhibition provides a target for immuotherapy in hematologic malignancies

The cell surface ATPase β subunit acts as a high-density lipoprotein (HDL) receptor, through binding of apolipoprotein A-I in hepatocytes, and also regulates lipoprotein internalization in endothelial cells [21]; however the effects downstream of the cell surface ATPase β subunit remain to be determined. ATPase β subunits have been detected on the membrane of tumor cells, raising the possibility that the structure of the β subunit protein on the cell surface may perform a different function to the inner mitochondrial protein structure. Our findings indicate that ectopic expression of the ATPase β subunit is a tumor-associated antigen in hematological malignancies. Although the function of the cell surface ATPase β subunit requires further study, this study implies that the ATPase β subunit plays an important role in cancer cell proliferation and apoptosis. Our findings are in agreement with previous studies which have indicated that angiostatin, plasminogen kringle 1–5 (K1–5), McAb against the ATPase β subunit [3,35] and small molecular inhibitors [1,36] can bind to ATP synthase on the cell surface and inhibit endothelial cell proliferation, migration, trigger apoptosis [3-6,10,14,19].

Cell surface ATP synthase is more active at a low extracellular pH [21]; therefore, ectopic expression of the ATPase β subunit may play an important role in the survival of cells suffering an energy shortage or during treatment with chemotherapy drugs, indicating cell surface ATP synthase may play important role in the development and treatment resistance of hematological malignancies. Our study suggests that abnormal cell surface expression of ecto-F1F0-ATPase β subunit may provide a potential target for cancer immunotherapy in hematological malignancies.

F1F0 ATP synthase was recently reported to be a co-chaperone of heat shock protein Hsp90, as F1F0 ATP synthase co-immunoprecipitates with Hsp90 and Hsp90-client proteins in cell lysate from MCF-7, T47D, MDA-MB-453 and HT-29 cancer cells [37]. Heat shock proteins are often overexpressed in human malignancies, including AML. Hsp90 is the major chaperone required for stabilization of the multiple oncogenic kinases involved in the development of AML [38]. Hsp90 client proteins are also involved in the regulation of apoptosis, proliferation, autophagy and cell cycle progression, and several hsp90 client proteins are considered to be possible therapeutic targets for the treatment of AML [39]. Hsp90 inhibitors could be used as single agents or potentially, in combination with other targeted treatments such as a functional ATP synthase β subunit antibody. This study indicates that clinical focus of hsp90 inhibitors and F1F0-ATP β subunit synthase functional antibodies should be directed towards hematological malignancies, as well as solid tumors and malignant melanoma.

## Conclusions

This study demonstrates that the β subunit of F1F0 ATPase is expressed on the cell surface of several leukemia cell lines with between 0.1% and 56% of cells expressing the ecto-F1F0-ATPase β subunit. We prepared a McAb against the ecto-F1F0-ATPase β subunit, which significantly inhibited proliferation and induced apoptosis in cell lines derived from AML *in vitro*. These findings indicate that expression of the ecto-F1F0-ATPase β subunit is a cancer-associated antigen in hematological malignancies. The ecto-F1F0-ATPase β subunit provides a potential target for immunotherapy in AML and other hematological malignancies.

## Abbreviations

ELISA: Enzyme-linked immunosorbent assay; McAb: Monoclonal antibody; AML: Acute myeloid leukemia.

## Competing interests

The authors have no conflicts of interest to disclose.

## Authors’ contributions

PJ designed and directed the study. ZWL, WJ and TYF finished the most of the experiments. FX and LYH drafted this manuscript. ZXM and ZM participated in the cell culture. NJ participated in study design and coordination, data analysis and interpretation and drafted the manuscript. All authors read and approved the final manuscript.
